# Inhibition of Interferon Induction and Action by the Nairovirus Nairobi Sheep Disease Virus/Ganjam Virus

**DOI:** 10.1371/journal.pone.0028594

**Published:** 2011-12-05

**Authors:** Barbara Holzer, Siddharth Bakshi, Anne Bridgen, Michael D. Baron

**Affiliations:** 1 Institute for Animal Health, Pirbright, Surrey, United Kingdom; 2 School of Biomedical Sciences, University of Ulster, Coleraine, County Londonderry, United Kingdom; Kantonal Hospital St. Gallen, Switzerland

## Abstract

The Nairoviruses are an important group of tick-borne viruses that includes pathogens of man (Crimean Congo hemorrhagic fever virus) and livestock animals (Dugbe virus, Nairobi sheep disease virus (NSDV)). NSDV is found in large parts of East Africa and the Indian subcontinent (where it is known as Ganjam virus). We have investigated the ability of NSDV to antagonise the induction and actions of interferon. Both pathogenic and apathogenic isolates could actively inhibit the induction of type 1 interferon, and also blocked the signalling pathways of both type 1 and type 2 interferons. Using transient expression of viral proteins or sections of viral proteins, these activities all mapped to the ovarian tumour-like protease domain (OTU) found in the viral RNA polymerase. Virus infection, or expression of this OTU domain in transfected cells, led to a great reduction in the incorporation of ubiquitin or ISG15 protein into host cell proteins. Point mutations in the OTU that inhibited the protease activity also prevented it from antagonising interferon induction and action. Interestingly, a mutation at a peripheral site, which had little apparent effect on the ability of the OTU to inhibit ubiquitination and ISG15ylation, removed the ability of the OTU to block the induction of type 1 and the action of type 2 interferons, but had a lesser effect on the ability to block type 1 interferon action, suggesting that targets other than ubiquitin and ISG15 may be involved in the actions of the viral OTU.

## Introduction

Nairobi sheep disease virus (NSDV) is a member of the genus *Nairovirus* within the family *Bunyaviridae* and causes acute hemorrhagic gastroenteritis in sheep and goats, with very high morbidity and mortality rates in susceptible animals [1]. It was originally isolated in Nairobi, Kenya in 1910 by inoculation of sheep with the blood of sheep suffering from acute gastroenteritis. NSDV was originally thought to be endemic only in East Africa; recent sequence data showed that the same virus can also be found in many places in India and Sri Lanka where it is called Ganjam virus (GV) [2]. Daubney and Hudson showed that NSDV is primarily transmitted in East Africa by the hard tick *Rhipicephalus appendiculatus,* and that animals that were bred in areas where this tick was prevalent were immune, but animals that were moved into such areas died in large numbers [3], [4]. The virus is therefore only of limited effect on stable populations, but can be a severe limitation on trade or attempts to improve stocks through introduction of new animals. There is no current vaccine. In India, the virus is found in a number of tick species, primarily *Hemaphysalis intermedia* [5]. Sheep and goats are the only known vertebrate hosts of NSDV/GV [6], [7], although one or two cases of human infection through needle-stick injury have been reported as leading to mild febrile illness [8], [9].

Nairoviruses are small, enveloped RNA viruses in which the genome consists of three segments of single stranded, negative sense RNA, designated Large (L), Medium (M) and Small (S) [10]. The S, M and L segments encode, respectively, the nucleocapsid protein (N), at least two envelope glyoproteins (Gn and Gc) and the viral RNA-dependent RNA-polymerase (L). The L segment is unusual among bunyaviruses, being extremely long (>12 kb), encoding a single protein of >450 kDa. The carboxyterminal half of this protein contains most of the polymerase motifs, while the amino-terminal part is largely of unknown function. The genus contains more than 30 different virus isolates, loosely grouped based on serum cross-reactivity and hemagglutination inhibition [11], since sequence data on all but a few of these viruses has been limited or missing until recently. The most important serogroups are the NSDV serogroup, which also includes Dugbe virus (DUGV) and Kupe virus, and the Crimean Congo hemorrhagic fever virus (CCHFV) serogroup, which contains CCHFV and Hazara virus, both human pathogens. CCHFV causes a severe disease in human beings, with a reported mortality rate of 3–30% [12]. The disease is very similar to that caused in sheep by NSDV infection and is characterised by haemorrhage, myalgia, and fever.

The first line of defence against virus infections is innate immunity. The key players are interferons (IFNs) and other cytokines that are rapidly produced in virus infected cells (Reviewed in [13]). Three major classes of IFNs are known. Type I IFNs comprise the largest group, with multiple distinct IFNα genes, one to three IFNβ genes and other genes (IFNω, -ε, -δ, -κ). The first two are induced directly in response to viral infection whereas the others play less defined roles. Type II IFN has a single member, namely IFNγ, which is secreted by activated T cells and natural killer cells rather than in direct response to virus infections. A third class of IFNs has been described recently that shares the same pathway to sense viral infection as type I IFNs and is also induced directly in response to viral infection [14], [15], [16]. After an infected cell senses a viral infection, IFNs are produced and released from the cell to induce an antiviral state in both itself and neighbouring cells. Both type I and type II IFNs bind to their cognate cell-surface receptors, thereby activating a signal-transduction pathway that triggers the transcription of several hundreds of genes [17]. These IFN-stimulated genes (ISGs) have either IFN-stimulated response elements (ISRE) or GAS (Gamma-activated sequence) elements in their promoter region. Type I IFNs such as IFNα can lead to transcription of ISGs with ISRE or GAS elements, whereas IFNγ can only induce ISGs with GAS elements (reviewed in [18]).

It seems that most viruses studied so far have developed a strategy to counteract the host innate immune system [13]. Two Nairoviruses (CCHFV, DUGV) have been shown to be inhibited by MxA, a protein induced by type I IFNs [19], [20]. Since the viruses are inhibited by IFN-induced proteins, viral virulence will depend at least in part on the ability of the virus to avoid or block the type I IFN response, and it is therefore very likely that Nairoviruses have developed tactics against this host defence mechanism. The current knowledge on how Nairoviruses manipulate the host innate immune system comes mostly from studies with CCHFV. Andersson et al. have shown that CCHFV showed a markedly delayed type I IFN response in cell culture, up to 48 hours after infection, possibly by interfering with the pathway that leads to activation of interferon regulatory factor 3 (IRF3) [21]. In addition they found that CCHFV is insensitive to IFNα treatment applied six hours post-transfection. One possible explanation for the delay in the IFN response is given by another study showing that CCHFV has developed a mechanism to remove the 5′-terminal triphosphate group from its genome segments, thereby avoiding retinoic acid inducible gene I protein (RIG-I)-dependant IFN induction [22]. Another possible explanation may be the activity of the ovarian tumour-like (OTU) domain found in the amino-terminus of the viral L protein. Sequence analysis of the L proteins of Nairoviruses identified this domain, which represents a unique class of deubiquitinating enzymes (DUBs) [23]. Recent studies with the CCHFV OTU-containing L protein showed that it decreases the coupling of ubiquitin (Ub) and the ubiquitin-like protein (Ubl) encoded by interferon stimulated gene 15 (ISG15) to cellular proteins [24]. The post-translational modification of proteins by Ub and Ubls regulates essential processes in the type I IFN response to viral pathogens [25], [26]. Cellular DUBs have been found which appear to act as part of negative feedback control systems for IFN induction pathways [27], [28]. Removal of Ub and/or ISG15 from their protein conjugates will disrupt a number of elements of the IFN induction pathway, and targeting the ISG15 and/or the ubiquitin system is a common strategy used by different viruses to inhibit innate immune responses [29], [30], [31], [32].

Up till now there is no information available on if and how NSDV/GV manipulates the host innate immune system. Because of the difficulties in handling CCHFV, there is only limited correlation of studies on virus and on viral proteins. We provide here the evidence that NSDV/GV is able to inhibit IFN induction as well as IFN action. We could identify the viral L protein as being responsible for these inhibitory effects. Furthermore NSDV is able to reduce total protein ubiquitination and ISG15ylation in infected cells. This deconjugating activity, as with CCHFV, is located in the N-terminal part of the NSDV L protein containing the OTU domain. Inactivation of the OTU enzymatic activity resulted in loss of its ability to antagonise IFN responses.

## Results

### Delayed IFNβ induction in NSDV-infected Vero cells

Two isolates of NSDV/GV were available to us for these studies, one a highly tissue-culture passaged isolate of the virus from Uganda (and therefore notionally NSDV), the other an isolate from India (and therefore notionally GV) which had been passaged a limited number of times in mouse brain or BHK 21 cells. Although these two isolates have been shown phylogenetically to be the same virus [2], we will refer to them as GV and NSDV in this paper to indicate the two isolates. The GV isolate proved to be still pathogenic in sheep, while the NSDV isolate was nearly completely attenuated, and the GV isolate is therefore probably nearer to wild type virus.

**Figure 1:**
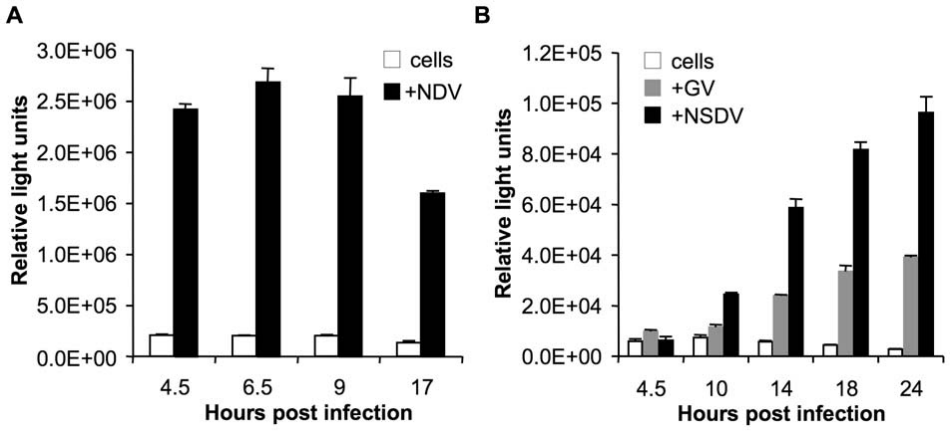
Delayed IFNβ induction in GV and NSDV-infected cells. Vero cells were transfected with 400 ng of pIFNβ-luc and 200 ng pJATLacZ. After 24 hours of transfection, the cells were infected with (a) NSDV or GV, or (b) NDV at an MOI of 1 TCID_50_ unit per cell, or left uninfected. At the indicated time points cells were lysed and assayed for luciferase and β-galactosidase activities. The ratio of these two activities was taken as the relative luciferase activity (in RLU). Shown are the data from a representative experiment in triplicates; error bars represent one standard error of the mean.

As described in the Introduction, studies with CCHFV have shown it has a delayed IFN response in infected cells [21]. We wanted to know if NSDV/GV is able to interfere with the early induction of type I IFNs. To address this question we transfected Vero cells with a reporter gene construct containing the firefly luciferase under the control of the IFNβ promoter. Luciferase expression is taken as a measure of protein production from this specific promoter after activation of transcription, which in turn is taken as a measure of IFNβ induction. Although this system is commonly used to study the control of IFN induction, we recognise that failure to express luciferase could reflect viral effects at a number of different points in the induction, transcription and protein synthesis pathway. As a positive control for the induction of IFNβ we used Newcastle disease virus (NDV), a paramyxovirus which has been reported as being unable to block IFN induction in mammalian cells [33] and therefore is frequently used as a model stimulator of cytoplasmic PRRs. Vero cells lack a functional IFN-β gene [34], [35] which made them a useful tool for our experiment because they allowed us to measure direct activation of the IFNβ promoter in infected cells excluding any indirect effect of IFN synthesis in neighbouring uninfected cells. The transfected Vero cells were infected with the NSDV or GV isolate or with NDV and the amount of synthesised luciferase was determined at the indicated time points (fig. 1a&b). Infection with NDV induced a rapid activation of the IFNβ promoter (fig. 1a); after 4.5 and 6.5 hours of NDV infection very high amounts of luciferase are already detected when compared to mock-infected cells, with levels of luciferase already decreasing at 9hpi. In contrast, at 4.5hpi there was no increase in promoter activity detectable in GV/NSDV-infected cells when compared to mock-infected cells (fig. 1b). A very slight increase in luciferase was seen at 10hpi in NSDV-infected cells, and at 14 hpi there is a clear significant increase in the reporter gene activity induced by NSDV or GV infection with further increases up to 24 hours of infection. Recent evidence suggests that many negative-strand RNA viruses do not generate dsRNA during infection [36] and only induce IFN rapidly if they contain defective interfering particles (DIs) [37]. It is likely therefore that our NDV preparation is acting through a significant content of such DIs. Neither NSDV/GV isolate, however, induced interferon until later stages in the infection cycle. There was a significant difference between the two isolates, NSDV and GV, regarding the kinetics of activation of the IFNβ promoter. Activation by the GV isolate was later than that induced by the NSDV isolate and also the transcriptional activation observed was lower when compared to the NSDV isolate (fig. 1b). This might be due to the fact that the NSDV isolate was previously passaged over 60 times in cell culture, in contrast to the GV isolate which has primarily been maintained in mouse brain culture and has been passed twice in BHK-21 culture in our hands; this difference may have resulted in the NSDV isolate having a reduced efficiency to evade IFN induction, or a more rapid growth in the cultured cells, leading to a more rapid production of one or more PAMPs recognised by the host cell. This second possible explanation is supported by the growth kinetics of GV and NSDV in Vero cells, which showed that NSDV replication rates were faster compared to GV, although both isolates grew to the same final titer (Lidia Lasecka, pers. communication).

### NSDV/GV is able to block transcription from the IFNβ promoter at early stages of infection

**Figure 2:**
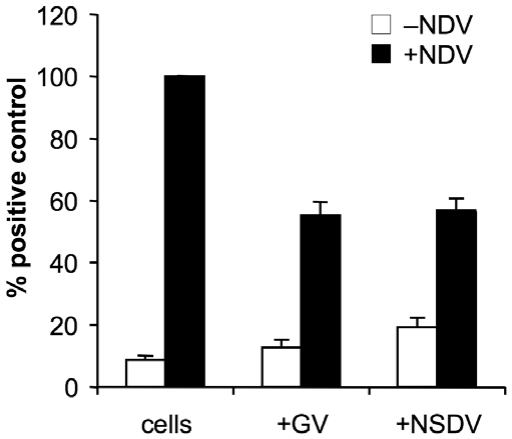
NSDV and GV suppress transcription from the IFNβ promoter. Vero cells were transfected with 500 ng each pIFNβ-luc and pJATLacZ. After 24 hours of transfection cells were infected with GV and NSDV at an MOI of 1 TCID_50_ unit per cell or left uninfected. Eight hours after infection with NSDV or GV, cells were superinfected with Newcastle disease virus (NDV) at an MOI of 1 TCID_50_ unit per cell or left uninfected, and finally lysed four hours after infection with NDV. The luciferase and β-galactosidase activities of the cell extracts were determined. Results from three separate experiments were combined by setting the RLUs induced by NDV in uninfected cells to 100%; all experiments were performed in triplicate wells. Error bars show standard errors of the normalised data.

Next we wanted to examine if the absence of IFNβ promoter activation in early stages of NSDV infection upon NSDV/GV infection is due to an active block or if NSDV/GV is rather avoiding the activation of the IFNβ promoter by a similar mechanism as already described for CCHFV [21]. On that account we transfected Vero cells with the IFNβ reporter gene plasmid. One day later cells were infected with the GV or NSDV isolate, and subsequently super-infected with NDV. Immunofluorescence experiments showed that approx 85% of cells were infected with NSDV/GV, and that prior infection with NSDV/GV did not block NDV infection in these cells (data not shown). The reporter gene activity was determined as described in material and methods (fig. 2). In cells infected with NDV alone the expected increase in the activity of the IFNβ promoter was observed when compared to uninfected cells. Interestingly, when the NDV infection was preceded by infection with GV or NSDV there was a clear reduction in the promoter activation detectable compared to cells infected solely with NDV. The reduction in the effects of NDV in these assays was only 40% even though about 85% of the cells were infected with NSDV/GV. This may have been because there was insufficient NSDV/GV protein at the time of the NDV superinfection to provide a complete block of the IFN induction pathway inside each infected cell; the reduction in reporter gene activity was indeed less pronounced if NDV was applied after shorter periods of GV or NSDV infection such as four hours post infection (data not shown), showing that it takes some time for the active block of the IFN induction pathway to take effect. This discrepancy may also reflect the limited nature of the block provided by NSDV/GV, which is unable to completely prevent activation of the IFN-beta promoter later in its own infection (FIg 1b), as well as the very strong stimulus provided by the NDV superinfection in these experiments. Nevertheless, these results indicate that NSDV and GV are able to actively suppress activation of the IFNβ promoter, and are not simply avoiding detection by cellular PRRs.

### NSDV/GV inhibits action of type I and II IFNs

**Figure 3:**
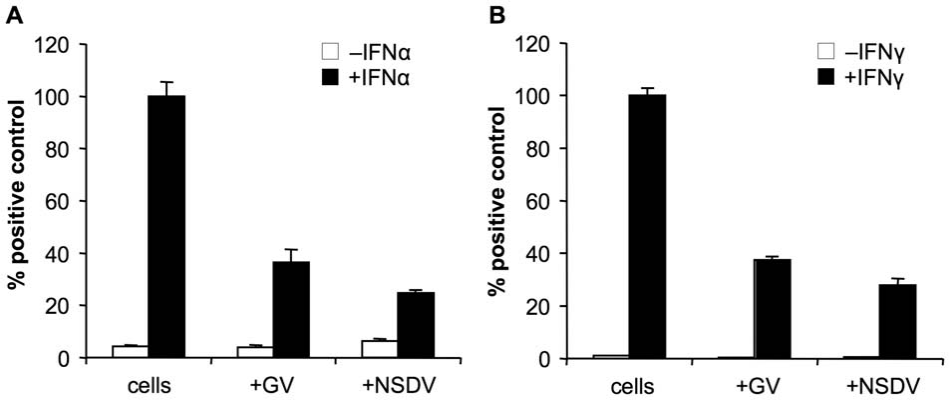
GV and NSDV inhibit the induction of expression from IFN-responsive promoters. Vero cells were transfected with 500 ng pJATLacZ and (a) 500 ng pGL3-Mx-1-luc or (b) pGAS-luc. 30 hours post transfection cells were infected with GV or NSDV at an MOI of 1 or left uninfected. 18 hours post infection cells were treated for 6 hours with IFNα (a) or IFNγ (b), lysed, and the luciferase and β-galactosidase activities determined. Results from two (for IFNα) or three (for IFNγ) separate experiments were combined by setting the RLUs induced by IFNα or IFNγ in uninfected cells to 100%; all experiments were performed in triplicate wells. Error bars show standard errors of the normalised data.

We wanted to know if NSDV is able to interfere with type I and type II IFN signalling. Vero cells were transfected with plasmids having the luciferase ORF under the control of the mouse Mx1 promoter (a promoter strongly activated by type I IFN) or one containing multiple copies of a GAS element (responds to type II IFN). Those cells were subsequently infected with either the GV or the NSDV isolate. After eighteen hours of infection cells were treated with IFNα or IFNγ or left untreated. Finally the luciferase activity was determined as described in material and methods. Treatment of cells with IFNα induced high levels of luciferase activity in cells transfected with the Mx-1 reporter plasmid (fig. 3a) which is in accordance with the literature [38]. Infection with either GV or NSDV resulted in a significant reduction in IFN-induced Mx-1 promoter activity when compared to uninfected cells. The same effect could be observed when cells were treated with IFNγ to induce the GAS element (fig. 3b). GV and NSDV were similarly effective in reducing the promoter activity in the presence of IFNγ to less than forty percent of the activity in IFNγ-stimulated uninfected cells. These data strongly suggest that NSDV/GV is able to counteract the type I and II IFN induced transcription of genes that have an ISRE or GAS element in their promoters.

### NSDV/GV inhibits the phosphorylation of STAT1 and STAT2 in type I and II IFN-stimulated Vero cells

**Figure 4:**
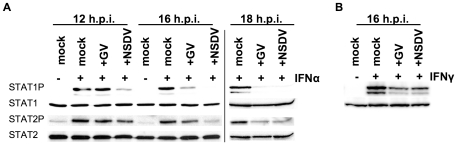
GV and NSDV infection interfere with the tyrosine phosphorylation of STAT1 and STAT2 in response to IFNα or IFNγ. Vero cells were infected with GV or NSDV at an MOI of 1 TCID_50_ unit per cell or left uninfected (mock). At the indicated time post-infection cells were treated with or without IFNα (a) or IFNγ (b) for 30 min. The cells were harvested and the levels of STATs and tyrosine-phosphorylated STAT proteins were determined by Western blot analysis with the corresponding specific antibodies. The data shown are from a representative experiment.

The binding of IFNα/β to the type I IFN receptor results in the autophosphorylation and activation of JAK (Janus activated kinase) 1 and tyrosine kinase 2, which are both members of the JAK family and associated with the receptor (reviewed in [18]). The activated JAKs phosphorylate specific tyrosines of STAT1 (signal transducer and activator of transcription 1) and STAT2. Upon phosphorylation, STAT1 and STAT2 form heterodimers and, in association with other factors, translocate to the nucleus to bind to ISREs to initiate transcription of ISGs. Binding of IFNγ to its receptor similarly induces phosphorylation of STAT1 which then forms homodimers that translocate to the nucleus to activate the transcription of genes with GAS elements. Targeting the activation of STAT proteins or their transfer to the nucleus are efficient ways to block innate immunity that are used by several different viruses (reviewed in [13]). To investigate whether NSDV/GV are inhibiting IFN action through an effect on STAT phosphorylation, we infected Vero cells with NSDV or GV for 16 hours and then stimulated the infected cells with IFNα or IFNγ. Samples from those cells were subjected to immunoblot analysis to check the phosphorylation status of STAT1 and STAT2 (fig. 4). In uninfected cells IFNα treatment induced phosphorylation of STAT1 and 2 (fig. 4a). An almost complete block of STAT1 phosphorylation was observed in NSDV infected cells, especially at later time points. The GV isolate also inhibited STAT1 phosphorylation, though was clearly less effective. This reduced effectiveness of the GV isolate corresponds to a reduced ability to block IFN action in the reporter gene assay (fig. 3), and may be due to the slightly slower growth of this isolate in cell culture; at later time points of infection (18hpi) the GV isolate was equally effective as the NSDV (fig. 4a). Interestingly neither virus was as effective at inhibiting the phosphorylation of STAT2 (fig. 4a). Treatment with IFNγ leads to the rapid phosphorylation of STAT1 in uninfected cells (fig. 4b). A clear reduction of phosphorylated STAT1 can be detected in NSDV- and GV-infected cells stimulated with IFNγ when compared to uninfected cells. No degradation of STAT 1 or 2 was observed in infected cells. This reduction in phosphorylation of the STAT proteins gives a possible explanation of the inhibitory effects of NSDV and GV on the GAS and ISRE promoter activity shown previously (fig. 3).

### The GV L protein inhibits transcription from the IFNβ promoter

**Figure 5:**
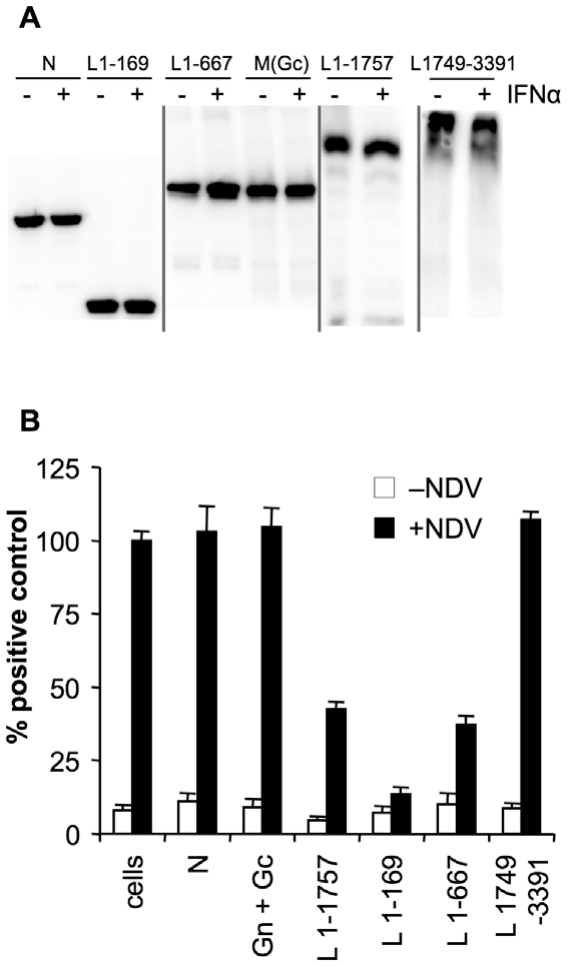
Ganjam L protein inhibits transcription from IFNβ-promoter in response to NDV infection. (a) Vero cells were transfected with 350 ng pIFNβ-luc, 200 ng pJATLacZ and 1.2 µg of a plasmid driving the expression of the indicated viral protein, with the exception of pcDNA6-GV-L1-169 and pcDNA6-GV-N where 700 ng and 300 ng respectively were used in transfection experiments. All transfections were made up to the same amount of DNA using empty vector. After 40 hours of transfection cells were infected with NDV (MOI = 1). After a further 5 hours the cells were (a) lysed with SDS-PAGE sample buffer and the expression of viral proteins detected by Western blot using a monoclonal antibody to the V5 epitope or to the His epitope as required, or (b) lysed with NP40 lysis buffer as described in Methods and the luciferase and β-galactosidase activities determined. Results from two or three separate experiments were combined by setting the RLUs induced by NDV in cells transfected with empty vector to 100%. Error bars show standard errors of normalised data.

So far the viral RNA-dependent RNA polymerase (RdRP) from CCHFV and DUGV are the only nairoviral proteins known to interfere with the host innate immune response [24]. We wanted to identify the NSDV protein(s) that is/are responsible for the inhibitory effects on IFN induction and action in infected cells. For that reason we made viral protein expression plasmids that were derived from the GV isolate as it is far less tissue culture adapted than the NSDV isolate. We cloned the open reading frame (ORF) of the S and M segments of GV into a mammalian expression vector under the control of a CMV promoter and with a V5-tag in frame at the C-terminus of each protein. For the M segment, which is translated into a polyprotein that produces at least two glycoproteins, this produces a V5 tag at the C terminaus of Gc, the most distal of the glycoproteins produced from this segment. We were unable to clone the complete ORF of GV (or NSDV) L into a plasmid due to recombination events that took place in *Escherichia coli*, even in strains such as STBL2, SURE2, ABLE K, XL-10 and MDS42 which are engineered to support unstable DNA. We mapped the toxic/unstable sequence to a region of approximately 1 kb found roughly in the middle of the L ORF. We were able to construct plasmids containing either half of this sequence but not the whole piece, and were therefore able to prepare expression constructs encoding the amino- and carboxy-terminal parts of GV L protein (aa L1-1757 and aa L1749-3391 respectively), thereby covering the whole protein with a short overlap between the constructs. In addition we cloned two shorter fragments of the amino terminal part of the viral polymerase that contained the OTU domain (aa L1-169) or the OTU domain and the following zinc-finger domain (aa L1-667). We transfected different amounts of these expression plasmids to approximately equalize the expression levels of the individual viral proteins in the reporter gene assays. In fig. 5a typical protein expression levels are shown that were used in our studies. Only the expression of the glycoprotein Gc which had the carboxy-terminal V5 tag could be analysed. The level of Gn could not be checked due to the lack of glycoprotein-specific antibodies for NSDV. However we assumed that the expression levels of the two glycoproteins are similar as they are expressed as a single polyprotein.

We wanted to know if one of these viral proteins is able to block the transcription from the IFNβ promoter in Vero cells infected with NDV. To address this question we co-transfected these viral protein expression constructs together with our reporter plasmids pIFNβ-luc and pJATLacZ into Vero cells. Then the transfected cells were infected with NDV and luciferase activity in cell extracts was analysed (fig. 5b). Neither the nucleoprotein nor the glycoproteins could impede NDV-induced transcriptional up-regulation of the IFNβ promoter. All three amino-terminal expression constructs containing the OTU domain of the GV L protein (L1-169, L1-667 and L1-1757) were able to significantly decrease luciferase expression, whereas the C-terminal part of L (L1749-3391), which contains parts of the polymerase but no OTU domain, showed no effects on NDV-induced reporter gene expression. The protein L1-169 was more effective than L1-667 and L1-1757 in blocking NDV-induced IFNβ-promoter activity. These data strongly suggest that the GV OTU domain is responsible for the inhibitory effects on type I interferon induction observed in infected cells (fig. 2).

### The OTU domain of the GV L protein blocks type I and II IFN action

**Figure 6:**
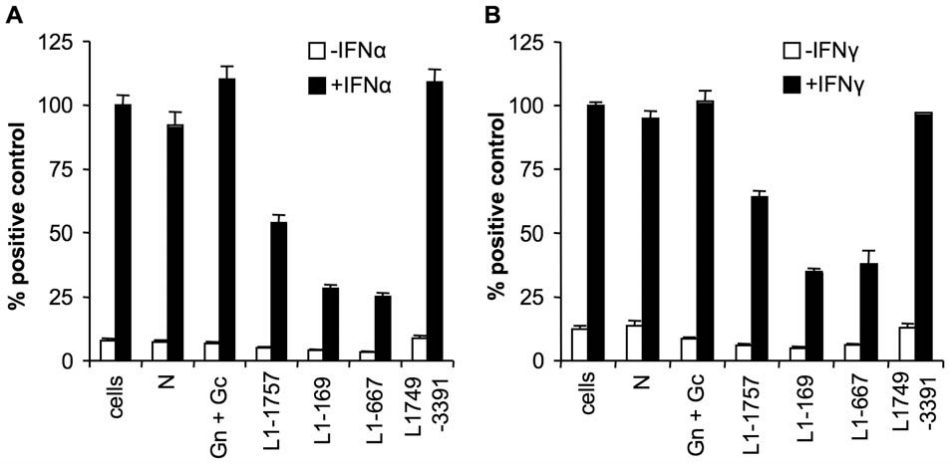
GV L protein inhibits transcription from IFN-responsive promoters. (a) Vero cells were transfected essentially as for Figure 5 except that 100 ng of the reporter gene plasmid pGL3Mx-1-lucwas used. All transfections were made up to the same amount of DNA using empty vector where required. After 40 hours of transfection cells were incubated with or without IFNα. After a further 8 hours the cells were lysed and the luciferase and β-galactosidase activities determined as described in material and methods. (b) Vero cells were transfected essentially as for Figure 5 except that 400 ng of the reporter gene plasmid pGAS-luc was used. 40 hours post-transfection cells were treated with IFNγ. After a further 6 hours cells were lysed and the luciferase and β-galactosidase activities determined. Results from separate experiments were combined by setting the RLUs induced by IFNα in cells transfected with empty vector to 100%. Error bars show standard errors of normalised data.

Our previous experiments have shown that NSDV is able to inhibit the action of type I and type II IFNs in infected cells (fig. 3). To identify the viral protein that is responsible for this effect we transfected Vero cells with reporter plasmids carrying a luciferase gene under the control of type I and II IFN-responsive promoters (pGL3-Mx-1-luc and GAS-luc) together with the viral protein expression plasmids. The cells were treated with IFNα or IFNγ to induce transcription from the IFN-responsive promoters and the luciferase induced was measured (fig. 6). The reporter gene activity in cells treated with IFNα or IFNγ was significantly reduced in the presence of the GV L protein constructs containing the OTU domain (L1-169, L1-667 and L1-1757) compared to cells transfected with empty plasmid only, whereas in cells expressing the nucleoprotein, the glycoproteins or the carboxy-terminal part of the L protein, treatment with IFNα or IFNγ induced comparable levels of reporter gene activity to that found in cells expressing no viral protein. As in the studies of IFN induction, the two shortest versions of the GV L protein were the most efficient in blocking the action of IFNα and IFNγ.

### Infection with NSDV/GV reduces total protein ubiquitination and ISG15ylation

**Figure 7:**
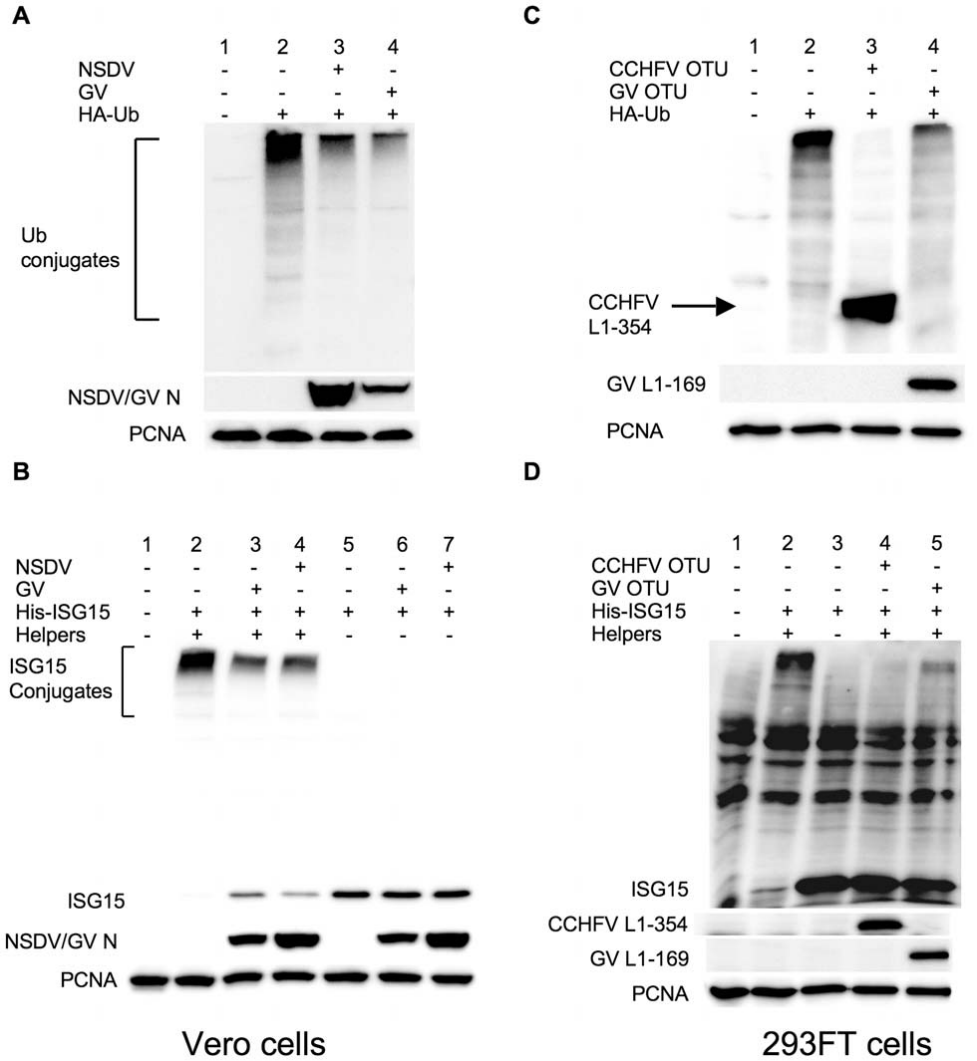
GV and NSDV inhibit ubiquitination and ISG15ylation. (a) Vero cells were transfected with 500 ng pHA-Ub. 14 hours post transfection cells were infected with NSDV or GV at an MOI of 1 TCID_50_ unit per cell. After a further 12 h cells were lysed in SDS PAGE sample buffer and total protein ubiquitination was analysed by Western blot using anti-HA. Lane 1: Untransfected cells; Lanes 2-4:transfected with pUb-HA; Lane 3: GV-infected; Lane 4: NSDV-infected. (b) Vero cells were transfected with 250 ng pHis-mISG15 with 750 ng empty vector (Lanes 5–7) or with 250 ng each HA-mHerc6, mUBE1L-HA and UbcM8 (Lanes 2-4) or left untransfected (Lane 1). After 14 hours of transfection cells were infected with GV (Lane 3, 6) or NSDV (Lane 4, 7) at an MOI of 1 TCID_50_ unit per cell or left uninfected (Lanes 1, 2, 5). Twelve hours after infection, samples were analysed by Western blot using anti-6His antibody. Samples were also probed for the presence of viral protein (N) to confirm infection. (c) 293FT cells were transfected with 400 ng pUb-HA and with 500 ng empty vector (Lane 2) or with 500 ng of plasmid expressing CCHFV L1-354-HA (Lane 3) or GV L1-169-V5 (Lane 4) or left untransfected (Lane 1). 30 hours post transfection cells were lysed and total protein ubiquitination was determined by Western blot using anti-HA. The expression of GV L1-169 was detected by using anti-V5. (d) 293FT cells were transfected with 250 ng each pHis-mISG15, HA-mHerc6, mUBE1L-HA, UbcM8 alone (Lane 2) or together with 250 ng of plasmid expressing CCHFV L1-354-HA (Lane 4) or GV L1-169-V5 (Lane 5) or left untransfected (Lane 1). As extra negative control cells were transfected with 250 ng pHis-mISG15 and 750 ng empty plasmid (Lane 3). After 30 h of transfection cells were lysed and total ISG15ylation was analyzed by Western blot using anti-6His. The expression level of GV L1-169 and CCHFV L1-354 were assayed by using anti-V5 and anti-HA respectively. PCNA levels served as loading control in all experiments.

Data from studies with CCHFV L protein showed that the OTU domain is enough to inhibit total protein ubiquitination and ISG15ylation in 293T cells [24]. We wanted to know if NSDV/GV has any effects on total protein ubiquitination and/or ISG15ylation in cells. We investigated this using Vero cells transfected with expression constructs for tagged forms of Ub or ISG15, with appropriate supporting plasmids as required for the ISG15 system [24], [39]. Cells were transfected with a plasmid expressing HA-tagged ubiquitin and subsequently infected with the GV or NSDV isolates. Twelve hours post-infection cell extracts were prepared and the expression of ubiquitin conjugated-proteins determined by immunoblotting (fig. 7a), showing that infection with either isolate caused a decrease in total protein ubiquitination compared to uninfected cells. In a similar way we studied the effects of NSDV/GV on ISG15ylation of proteins in infected host cells. ISG15 is a Ubl that is very rapidly induced in type I IFN-treated cells [40] and is thought to play important roles in anti-viral responses, either as a monomer or by conjugation to host cell proteins (reviewed in [41]). ISG15 conjugation to host cell proteins exerts antiviral activity against influenza virus [42] and Sindbis virus [43]. For our studies on NSDV and its effects on host ISGylation during infection we made use of the fact that ISG15ylation can also be generated by transfecting expression constructs for ISG15 along with plasmids encoding the core components of the ISG15 conjugation system, the E1 activating protein (mUBE1L), E2 conjugating protein (UbcM8) and E3 ligase (mHerc6) into cells in the absence of IFN [39]. After transfecting these four plasmids into Vero cells, the cells were subsequently infected with the GV or NSDV isolates or left uninfected. Twelve hours post-infection cells were lysed and the level of ISG15-conjugates were examined by immunoblotting (fig. 7b). Infection of NSDV/GV resulted in a drastic decrease in ISG15-conjugates found in Vero cells when compared to uninfected cells. Importantly, the infection with NSDV itself did not cause a reduction in the total amount of mono-ISG15 expressed from the transfected plasmid, as shown by tracks 5-7 of fig. 7b, where the helper plasmids were omitted, so one is simply comparing ISG15 expression in uninfected and infected cells. This excludes any indirect effect of infection on ISGylation through an effect on ISG15 expression or stability.

Since the virus itself reduces Ub and ISG15 coupling to cell proteins, we wanted to confirm that the OTU domain is responsible for these decreased levels of conjugates in NSDV/GV as it is for CCHFV. For these studies we used 293 cells, as those were the cells used in the studies on CCHFV proteins. We determined the level of ubiquitination in the presence of the GV L1-169 protein in 293FT cells and compared it to the level found in cells transfected with empty plasmid or with the CCHFV L1-354 protein (containing the OTU domain of CCHFV) (fig. 7c). The CCHFV OTU-containing protein contains an HA tag and so appears in the same blots as the HA-Ub and HA-Ub conjugates. The CCHFV OTU completely abolished ubiquitination as previously shown [24]. The GV OTU was also effective in decreasing the levels of cellular Ub-conjugates, but was less effective compared to the CCHFV OTU domain. In the same way we investigated whether the OTU domain of NSDV/GV was responsible for the reduction in ISG15ylation. For this purpose we transfected 293FT cells with the core components of the ISG15 system in conjunction with plasmids expressing either the GV or CCHFV OTU domain or empty plasmid (fig. 7d). Expression of the GV OTU domain efficiently blocked conjugation of ISG15 to cellular proteins. The CCHFV OTU also blocked ISG15ylation in 293FT cells as already published [24]. Again the CCHFV OTU was more efficient than the GV OTU in reducing the amounts of ISG15-conjugated proteins in the cell. Both virus isolates of NSDV are therefore able to decrease total cellular ubiquitination and ISG15ylation levels during infection. The GV OTU domain, when expressed alone, could reproduce these effects exerted by the virus during infection.

### The catalytic activity of the OTU domain is necessary but not sufficient for antagonising IFN action

**Figure 8:**
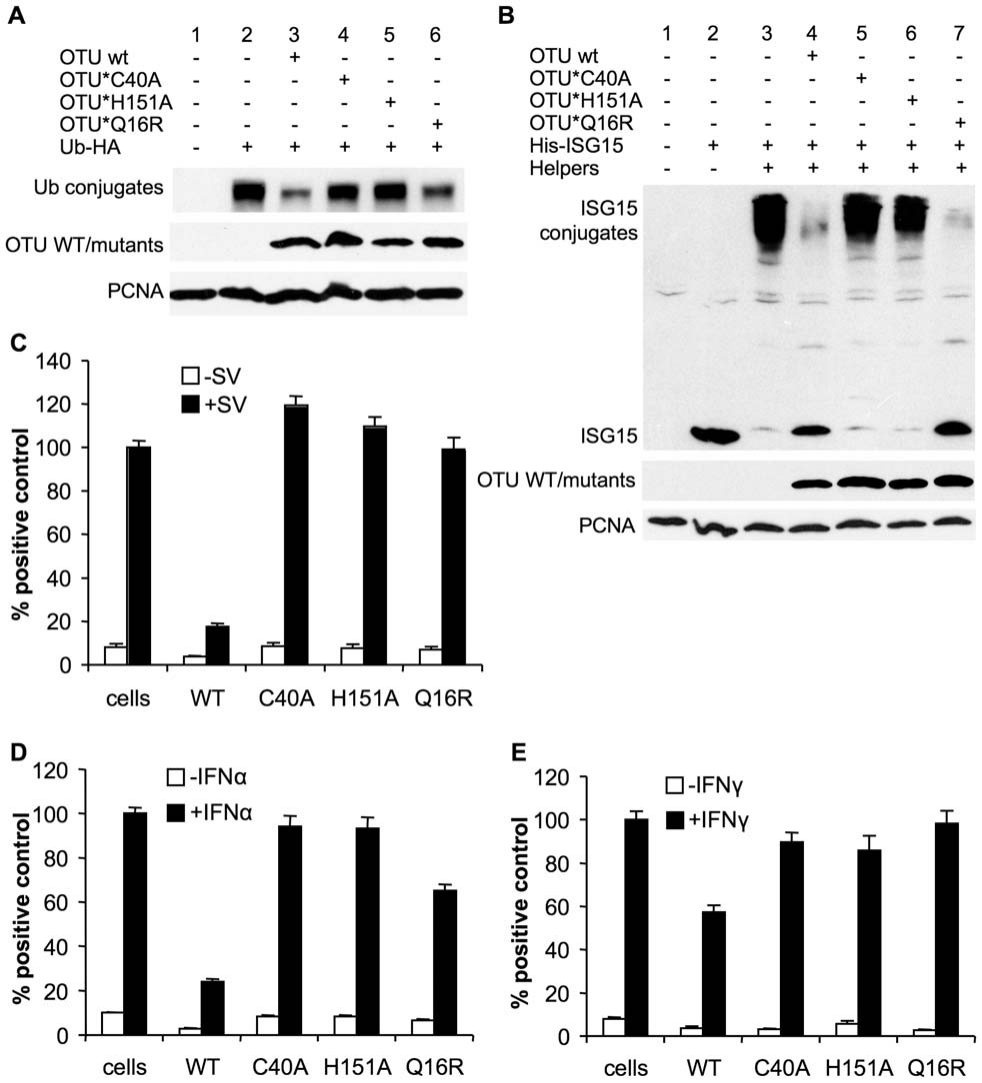
The OTU enzymatic activity is required for inhibition of IFN induction and action. (a) 293FT cells were transfected with 400 ng pHA-Ub and with 500 ng empty vector (Lane 2) or with 500 ng of plasmid expressing GV L1-169-V5 wildtype (Lane 3) or with the mutants C40A (Lane 4), H151A (Lane 5) or Q16R (Lane 6) or left untransfected (Lane 1). Thirty hours post-transfection, cells were lysed and total protein ubiquitination was determined by Western blot using anti-HA. (b) 293FT cells were transfected with 250 ng each pHis-mISG15, HA-mHerc6, mUBE1L-HA, UbcM8 alone (Lane 3) or together with 500 ng of plasmid expressing GV L1-169-V5 (Lane 4) or with one of the following mutants C40A (Lane5), H151A (Lane 6) or L16R (Lane 7), or left untransfected (Lane 1). As an extra negative control, cells were transfected with 250 ng pHis-mISG15 and 1250 ng empty plasmid (Lane 2). After 30 h of transfection cells were lysed and total ISG15ylation was analyzed by Western blot using anti-6His. The expression level of GV L1-169 and its mutants were assayed by using anti-V5. PCNA levels served as loading control in all experiments. (c) Vero cells were transfected with 350 ng of the reporter plasmid pIFNβ-luc plus 200 ng pJATLacZ combined with 700 ng of pcDNA6-GV-L1-169 or of one of the mutants C40A, H151A, or Q16R, or empty vector. After 24 hours of transfection cells were infected with Sendai virus (SV) or left uninfected. Eight hours post-infection the cells were lysed with NP40 lysis buffer and the luciferase and β-galactosidase activities determined as described in material and methods. (d) Vero cells were transfected essentially as in (c) except that 100 ng of the reporter plasmid pGL3Mx-1-luc was used. After 24 hours of transfection cells were incubated with or without IFNα. After a further 8 hours the cells were lysed and the luciferase and β-galactosidase activities determined as described in material and methods. (e) Vero cells were transfected essentially as in (c) except that 400 ng of pGAS-luc was used as the reporter plasmid. After 24 hours of transfection cells were incubated with or without IFNγ. After a further 8 hours the cells were lysed and the luciferase and β-galactosidase activities determined as described in material and methods. Error bars in (c-e) show standard error of the mean of normalised data. Results from three separate experiments were combined by setting the RLUs induced by SV or IFN in cells transfected with empty vector to 100%.

To determine the role of the catalytic activity of the OTU domain in IFN antagonism, we changed the cysteine at position 40 and the histidine at position 151, two components of the catalytic triad, to alanine (C40A and H151A respectively). A third mutant was created where glutamine 16 was replaced by arginine (Q16R); Q16 has been described as important for the binding of the CCHFV OTU to ubiquitin but with little importance for the binding of ISG15 [44], [45], so this mutation was designed to allow us to study solely the effects of deISG15ylation on the innate immune system. The mutations were introduced individually into the GV L1-169 construct, and transfected into 293FT cells to examine their effect on ubiquitination and ISGylation (fig. 8a, b). Mutation of C40 or H151 resulted in a complete loss of deubiquitinating (fig. 8a) and deISG15ylating (fig. 8b) activity. The Q16R mutant performed like the wildtype regarding its ability to remove ISG15 from cellular substrates (fig. 8b) while its deubiquitinating activity was only slightly reduced when compared to the wildtype OTU (fig. 8a). In previous studies on CCHFV a similar mutant was described as being unable to hydrolyze a fluorogenic model DUB substrate (Ub-AMC) [44]. The difference might be explained by the fact that we are using the OTU domain from a different virus and also a different assay for DUB activity.

We tested these mutants regarding their ability to interfere with IFN induction. For this purpose, cells were transfected with L1-169 or the catalytic mutants C40A, H151A or Q16R, along with the reporter plasmids pIFNβ-luc and pJATLacZ (fig. 8c). The IFNβ promoter was activated by infection of cells with Sendai virus (SV) for eight hours. Interestingly all three mutants lost their ability to block SV-induced IFNβ promoter activity when compared to the wildtype. These results showed that the catalytic activity of the OTU is necessary to counteract IFN induction. The results with the Q16R mutant were interesting, as it retained its deubiquitinating and deISG15ylation activity but, despite that, was not able to block IFN induction.

We also examined the ability of L1-169 C40A, H151A, and Q16R to block type I (fig. 8d) and II (fig. 8e) IFN action. We used the same experimental setup as described before when we tested the different viral proteins for their ability to block IFN action (fig. 6). The catalytic site mutants of L1-169 no longer blocked type I IFN-induced gene expression (fig. 8c). The Q16R mutant did block the induced transcription from the Mx-1 promoter to some extent, but not as efficiently as the wildtype. All mutants lost their ability to block type II IFN-induced gene expression (fig. 8d). These data show that the enzymatic activity of the OTU domain in the nairovirus L protein plays a pivotal role in antagonizing IFN induction and action. In addition, the data from the Q16R mutant strongly suggests that specific, yet different, targets are involved in antagonising these three activities, since this protein is still able to remove ubiquitin and ISG15 from the dominant cellular substrates, but lost its ability to antagonize IFN induction and type II IFN action while retaining some ability to block type I IFN action.

## Discussion

NSDV is regarded as one of the most pathogenic diseases in sheep and goats with mortality rates ranging from 40% in Merino sheep to 90% in Masai sheep [1]. Judging by its high pathogenesis, NSDV has most likely developed efficient mechanisms to circumvent or inhibit innate immunity. The first response of the immune system against virus infections is the production and secretion of type I IFNs. We could show that the induction of transcription from the IFNβ promoter in infected cells by NSDV/GV is delayed and reduced when compared to another negative strand RNA virus (fig. 1). We do not observe the extensive delay described previously in CCHFV infected cells [21]; however, that observed in our studies would give the NSDV enough time to establish its infection and produce progeny virions (which takes approx 12 hours in Vero or BHK21 cells).

A possible reason for the delayed induction could be a reduced production of PAMPs by the virus, such as the removal of the 5′ triphosphate from progeny viral genome transcripts, as observed for CCHFV [21]. Interestingly our results showed that NSDV is able to actively suppress the induction of IFNβ in infected cells, as both isolates were clearly able to reduce the NDV-induced transcription from the IFNβ promoter by approximately 40% (fig. 2) when assayed at 8-12hpi, before either NSDV/GV isolate showed strong induction of IFN.

Expression of the N-terminal part of the RNA-dependent RNA polymerase (L), which contains the OTU domain, was sufficient to reproduce the antagonistic effect on IFN induction in a reporter gene-based assay (fig. 5), and we could show that mutations that affected the catalytic site of the OTU protease were no longer antagonists (fig. 8). Ubiquitination and modification of cellular proteins with ubiquitin-like molecules such as ISG15 play important roles in regulating IFN induction through both the Toll-like receptor (TLR) and RIG-I-like receptor (RLR) pathways [46], [47]. Lys-63-linked polyubiquitination of RIG-I has been shown to be crucial for its ability to induce type I IFNs [48]. Arimoto et al. [49] showed that virus-induced IRF3 and NF-κB activation is dependent on the polyubiquitination of the protein NF-κB essential modulator (NEMO). Furthermore NF-κB activation is known to depend on ubiquitination of the inhibitor protein I-κB, targeting it for degradation [50], while ISG15ylation enhances NF-κB activity by conjugating to and suppressing protein phosphatase 2Cβ, which suppresses dephosphorylation of I-κB [51]. ISG15ylation positively regulates IRF-3 activation by preventing its interaction with PinI [52]. Wholesale removal of conjugated ubiquitin and ubiquitin-like modifiers would therefore greatly inhibit the IFN induction pathway. Indeed we observed a significant reduction in the levels of cellular ISG15- and ubiquitin-conjugates during NSDV/GV infection (fig. 7a, b) which could be attributed to the OTU domain of the L protein (fig. 7c, d). The enzymatic activity proved to be essential for the observed inhibition of IFN induction (fig. 8e).

Several other viruses have exploited this mechanism of negatively regulating the IFN induction by encoding proteases of the OTU family [24], [30], [53], [54]. All these proteases exert deconjugating activities towards ubiquitin or the ubiquitin-like (ubl) molecule ISG15 or both. Interestingly, cells themselves make use of DUBs to regulate the IFN pathway but, in contrast to nairoviral OTUs, cellular DUBs tend to have highly specific targets [27], [55], [56], [57]. For example, deubiquitinating enzyme A (DUBA) selectively cleaved polyubiquitin chains on tumor necrosis factor receptor-associated factor 3 (TRAF3) and was identified in a small interfering RNA-based screen as a negative regulator of type I IFN production [27]. The crystal structure of the CCHFV OTU domain revealed a unique structure allowing it to bind ISG15 as well as ubiquitin, and this ability to interact with both conjugating proteins is one of the underlying reasons for the promiscuous activity of the nairovirus OTUs [44], [45], [58].

We also found that NSDV is able to inhibit the action of type I and II IFNs, and that this activity involves the inhibition of phosphorylation of both STAT1 and STAT2. The mechanism(s) of this inhibition remains to be determined. There is no reduction in the levels of STAT1/2, and an inhibition of their phosphorylation suggests either binding/sequestration of one or both STATs or inhibition of the IFN receptor-associated JAKs. We observed that IFNγ-induced STAT1 phosphorylation was blocked similarly by both isolates at all times post infection, whereas IFNα-induced STAT1/2 phosphorylation was dependent the degree of growth of the virus, the block developing more slowly in cells infected with the slower-growing GV isolate. This suggests that the mechanisms by which the virus exerts the blockade of type I and 2 IFNs are not the same. Further evidence for this came from the GV L1-169 Q16R mutant, which retained most of its DUB activity and its full deISG15ylating activity and still shows significant blockade of IFNα-induced gene expression but no longer blocks IFNγ-induced gene expression (fig. 8c, d). It could be that this part of the N-terminus contains a specific-substrate binding site in addition to the catalytic core of the OTU domain, contributing to the effect of the OTU domain on specific cellular substrates; in this case the Q16R mutation exerts a steric inhibition on binding to the target involved in blocking type II IFN action, rather than an inhibition which is dependent on the OTU enzymatic activity. For the herpesvirus-associated ubiquitin-specific protease (HAUSP or USP7), an additional binding site with affinity for its target protein has been described in addition to its catalytic protease domain [59].

The virus had a stronger effect on IFNα-induced STAT1 phosphorylation than on STAT2 phosphorylation at all times, suggesting that STAT1 is the primary target. As with the block of IFN induction, the blockade of both type I and type II IFN actions mapped to the OTU domain of the L protein and required a functional OTU catalytic site. So far no definitive role for ubiquitination or ISG15ylation in type I or type II IFN signalling has been shown. JAK1 and STAT1 have been shown to be conjugated by ISG15 [60], [61]; however, STAT1 phosphorylation in response to IFN is normal in cells from ISG15 knock-out mice [62], suggesting that ISG15 plays no role in the immediate cell response to IFN.

Differences were seen in the effectiveness of the different OTU-containing fragments of the GV L to block IFNα- or IFNγ-induced gene transcription when expressed in our reporter gene-based studies. GV L1-169 and L1-667 reduced the transcriptional activity induced by IFNα or IFNγ to 30% and 40% respectively of the positive control, whereas the protein L1-1757 was less effective in reducing the transcriptional activity of the Mx-1 promoter or the GAS promoter (55% and 65% of the positive control respectively). These differences might reflect slight differences in the amounts of protein expressed in the transfected cells that cannot be detected by immunoblotting. Alternatively, the catalytic domain of the NSDV L protein might be autoinhibited by folding or oligomerisation in the longer construct L1-1757, in contrast to the shorter versions containing the OTU domain. A similar observation was made with the OTU domain containing non-structural protein 2 (nsp2) from porcine reproductive and respiratory syndrome virus where a longer fragment was less effective in blocking NFκB promoter activity than a smaller version of this protein [30]. In addition it has been shown that CCHFV full length L displayed significantly less DUB activity than shorter versions of the protein [24]. Further crystal structures to extend that of the basic OTU domain [44], [45], [58] will clarify these points.

One important factor that needs to be examined is the relationship of the reactivity of these OTU domains to species specificity of the viruses. Influenza B was found to inhibit the human but not the mouse ISG15ylation system [39]. The nairovirus OTUs appear to act by cleaving Ub and ISG15 from their respective conjugates, since mutations in the active site abolish activity [24], and the OTUs are therefore active against both human and murine systems. However, there was a clear difference between the activity of the CCHFV and NSDV/GV OTUs in the murine ISG15ylation system used in this study. We know little of the ruminant equivalents of the Ubls, and it is possible that the OTUs of different viruses may be adapted to species differences in these modifying proteins which could lead in turn to differences in the species specificity of pathogenesis. It will be important to examine the actual level of modification of host cell proteins when these viruses are grown in cells from different hosts to establish whether there is any degree of correlation between OTU cleavage activity and host cell species.

Within this study we could demonstrate that NSDV/GV is able to block the innate immune system at three different stages, type I IFN induction, type I IFN action and type II IFN action. NSDV/GV seems to be another example of a pathogen that exploits the host cell ubiquitin pathways for its own good by encoding an enzyme with deubiquitinating and deISG15ylating activity. However, to fully clarify the role of the OTU activity, an OTU knock-out virus is needed to evaluate the importance of the OTU domain *in vivo*, and we are working towards such a system. An advantage of NSDV/GV will be the ability to carry out such experiments in the natural host.

## Materials and Methods

### Cells and Viruses

The Vero cells (African green monkey kidney cells) used in these studies were a modified line that expresses CD150 (aka Signaling Lymphocyte Activation Molecule (SLAM), as these were the Vero cells in use in our group for other studies on morbilliviruses. They are otherwise identical to normal Vero cells and are referred to as Vero cells throughout. This line was obtained from Dr Rick De Swart, Erasmus Medical College, The Netherlands and was maintained in Dulbecco's Modified Eagle's Medium (DMEM) with 25 mM HEPES buffer supplemented with 5% foetal calf serum (FCS), penicillin (100 U/ml) and streptomycin sulphate (100 µg/ml). 293FT (a fast-growing variant of the HEK293 human embryonic kidney cell line that expresses the SV40 large T antigen) (a kind gift of Prof R. E. Randal, St. Andrews University, Scotland) were maintained in DMEM with 25 mM HEPES buffer supplemented with 10% foetal calf serum (FCS), penicillin (100 U/ml) and streptomycin sulphate (100 µg/ml). BHK21 clone 13 (baby hamster kidney) cells (kindly provided by Dr T. Jackson, Institute for Animal Health, Pirbright, UK) were cultured in Glasgow Modified Eagle's Medium (GMEM) containing 10% FCS, penicillin (100 U/ml), streptomycin sulphate (100 µg/ml), 2 mM L-glutamine and 5% tryptose phosphate broth solution (Sigma).

The Nairobi sheep disease virus (NSDV) isolate (ND66-PC9) was obtained from Dr Piet van Rijn, Central Veterinary Institute of Wageningen, Netherlands. The Ganjam virus (GV) isolate (IG619, TVPII 236) was obtained from World Reference Center for Emerging Viruses and Arboviruses at the Galveston National Laboratory, and was the kind gift of Prof Robert B Tesh, University of Texas Medical Branch, Galveston, Texas, USA. Virus stocks were grown in BHK21 cells using GMEM containing 2% FCS, penicillin (100 U/ml), streptomycin sulphate (100 µg/ml), 2 mM L-glutamine and 5% tryptose phosphate broth solution. The virus titre was determined as the 50% tissue culture infectious dose (TCID_50_) in BHK21 cells. Both strains grew to similar final titres (∼10^6^/ml). Multiplicity of infection (MOI) was calculated as TCID_50_ per plated cell. The Ulster 2C vaccine strain of Newcastle disease virus (NDV) (grown in eggs) was the kind gift of Prof W. Barclay, Imperial College, London, UK. The NDV titre was determined as the TCID_50_ in Vero cells. The Sendai virus Cantell Strain (ATCC VR-907 Murine parainfluenza virus type 1) was purchased from Charles River Laboratories, USA.

### Plasmids and antibodies

Except where indicated, all DNA manipulation was done following standard methods. Plasmids were cloned and grown in *Escherichia coli* DH5α or SURE2 (Stratagene). Routinely plasmid DNA was purified on CsCl gradients. The plasmids pJAT-lacZ, pGAS-luc, and pIFNβ-luc were the kind gifts of Prof Steve Goodbourn, St. George's Hospital Medical School, London, United Kingdom. The pGL3-Mx1P-luc was kindly provided by Prof Georg Kochs, Department of Virology, University of Freiburg, Germany. The following plasmids were used for the ISG15ylation and ubiquitination experiments: pCAGGS.MCS-6HismISG15, and plasmids expressing mHerc6, Ubcm8 and mUbE1L were provided by Prof Deborah J. Lenschow, Washington University School of Medicine, St. Louis, Missouri. Plasmid pHA-CCHFV-L1-354 is in pCAGGS-MCSII and was the gift of Dr Natalia Frias-Staheli, Mount Sinai School of Medicine, New York.

#### Construction of viral protein expression plasmids

Total RNA from GV-infected BHK21 cells was extracted by using RNeasy Mini Kit (Quiagen) which served as a template for cDNA synthesis using random priming oligos and SuperscriptII Reverse Transcriptase (Invitrogen). The viral genome was amplified by PCR using NSDV/GV genome-specific oligos and subsequently blunt-end cloned into pT7Blue (Novagen). All PCRs were performed using proofreading polymerase (KOD; Novagen). The genome of both strains was completely sequenced. Plasmids pcDNA-GV-N and pcDNA-GV-M were made by cloning the complete ORFs of GV S and GV M segments into pcDNA6/V5-His (Invitrogen), expressing C-terminal V5-tagged N and the glyoproteins (only Gc has a V5 tag at its C-terminus). GV L1-169 and L1-1757 were cloned in pcDNA6 with a C-terminal V5 tag. GV L1-667 and L1749-3391 were cloned into pTriEX (Novagen) such that the expressed proteins have a 6xHis tag at the C- terminus. To generate catalytically inactive variants of L1-169 in pcDNA6/V5-His, single amino acid mutations were introduced by overlap PCR mutagenesis. All mutations were confirmed by sequencing the entire open reading frame.

Mouse monoclonal antibody against phosphotyrosine 701-STAT1 were purchased from BD Biosciences. Polyclonal antibodies against STAT1, STAT2, and phosphotyrosine 689-STAT2 were obtained from Upstate. Mouse monoclonal antibody against proliferating cell nuclear antigen (PCNA) was obtained from Santa Cruz Biotechnology. Mouse monoclonal anti-His antibody was purchased from Sigma Aldrich and HRP-tagged anti-HA antibody from Roche. The rabbit anti-N antiserum recognizing the amino terminus of the NSDV and GV N protein were previously made in our laboratory. Mouse monoclonal antibody against the V5 epitope tag was purchased from AbD Serotech. Mouse monoclonal antibody U85 recognising Newcastle disease virus was the kind gift of Ruth Manvell, AHVLA, Weybridge, UK.

### Transfections and reporter gene assays

All transfections were carried out with TransIT LT1 (Mirus) according to the manufacturer's instructions. A ratio of 2 or 3 µl LT1 per µg DNA was used. Cells were plated at 10^5^ per well in 12-well plates 1 day before use. Usually, 24 hours post transfection the medium containing the transfection mix was removed and replaced with fresh medium. The cells were then lysed in 200 µl lysis buffer (120 mM NaCl, 50 mM TrisCl pH 7.5, 0.05% Nonidet P-40). The samples were centrifuged for 1 min at full speed in a table top centrifuge. 50 µl of the cleared cell extract was taken and the luciferase activity was measured after adding 50 µl of luciferase assay reagent (Promega) to each sample. The following settings were used to measure the relative light units: integration time: 10 sec, sensitivity: 200 or 230 and Filter set 1. To determine the β-galactosidase activity 150 µl of assay buffer (48 mM Na_2_HPO_4_, 32 mM NaH_2_PO_4_, 8 mM KCl, 0.8 mM MgSO_4_, 3.2 mg/ml o-Nitrophenyl β-D-Galactopyranoside) were added to the samples after the luminescence was measured. The samples were incubated at 37°C for 30 min to 60 min before absorbance at 420 nm was measured. The luminescence and absorbance measurements were done in a Synergy 2, Multi Detection Microplate Reader (BioTek Instruments) using Gen5 software. The values were normalised and statistically analysed as previously described [63].

### Virus infection, IFN treatment, and immunoblotting

Vero cells were plated at an initial seeding density of 1×10^5^/well in 12-well plates. One day later cells were infected with GV or NSDV at a multiplicity of infection (MOI) of 1. The virus inoculum was removed one hour after infection, cells were washed once with PBS and fresh medium was added. The infected cells were further incubated for 14 h before being treated with 1000 IU/ml recombinant human αA-Interferon (IFN) or 1000 IU/ml recombinant human IFN-γ. IFN-αA was purchased from Calbiochem and IFN-γ was obtained from Millipore. Cells were treated for 30 min with or without IFN before being harvested and lysed with 100 µl of 1x SDS sample buffer (New England Biolabs). SDS-PAGE and Western blots were carried out as previously described [64].
